# Isolated and multiple causes of equine dystocia

**DOI:** 10.1186/s13028-024-00772-8

**Published:** 2024-10-11

**Authors:** Markus Ellerbrock, Judith Krohn, Kathrin Büttner, Axel Wehrend

**Affiliations:** 1 Veterinary Clinic for Reproductive Medicine and Neonatology, Justus Liebig University, Frankfurter Strasse 106, 35392 Giessen, Germany; 2 Unit of Biomathematics and Data Processing, Faculty of Veterinary Medicine, Justus-Liebig- Universität Giessen, Frankfurter Strasse 95, 35392 Giessen, Germany

**Keywords:** Dystocia causes, Foal mortality, Mare mortality

## Abstract

**Background:**

Dystocia is rare in horses, but is life-threatening for mares and foals. Therefore, veterinary research depends on up-to-date data to optimise equine dystocia management. In addition, knowledge of the prognosis of equine dystocia is necessary to advise animal owners. This retrospective study of equine dystocia aimed to enrich existing datasets with up-to-date information. For the first time, the focus was on the causes of dystocia that occurred alone or in combination. Over a period of 10 years, 72 cases of dystocia were analysed using a standardised, predetermined diagnosis code.

**Results:**

Of the 72 cases of dystocia, an isolated cause of dystocia was identified in 37 cases (51.4%) and 35 mares showed a combination of two or more causes (48.6%). Foetal causes were significantly more frequent origin of dystocia (66/69) than maternal causes (3/66) (*P* < 0.0001). Incorrect posture of the foetal forelimbs and head was the most common combination at 25.7%. The most common isolated cause of dystocia was incorrect posture of the foetal forelimbs (18.9%). A foetotomy was performed in 68% of cases. A caesarean section or an extraction was performed in 13.9% of the cases. Three mares died before any obstetric care was provided. Nine mares (12.5%) were not discharged alive. 73 foals were delivered of which 55 were already dead before veterinary treatment began. In relation to the total number of births in which the foetus was alive at the start of obstetric care, the foetal mortality rate was 61.1% and 88.9% by the time the mare was discharged.

**Conclusions:**

It has been demonstrated for the first time that multiple causes of dystocia in horses are almost as common as isolated causes of dystocia. Neonatal mortality remains high, indicating that the timely detection and treatment of dystocia has the highest priority.

## Background

Mares and foals are at serious risk of dystocia [1–3]. Despite advances in veterinary medicine, dystocia in horses is characterized by a relatively high mortality rate [4–6]. In particular, a short duration of dystocia and rapid veterinary intervention is necessary to avoid serious health consequences for the mare and foal [7, 8]. In the available literature, there are a number of case studies on the causes of equine dystocia [9–11]. However, no studies have differentiated between the occurrence of isolated and multiple causes (more than one) of dystocia.

Using a standardised diagnostic code, this study from an obstetric clinic aimed to provide updated information on the diagnosed isolated and multiple causes of dystocia and their consequences. Mortality rates and complications were analysed according to the different obstetric procedures. These findings contribute to the current knowledge of equine dystocia.

## Methods

### Animals

Data were collected and analysed from mares that presented to the Veterinary Clinic for Reproductive Medicine and Neonatology at the Justus Liebig University, Germany in the period 01.01.2010 to 31.12.2020 because of dystocia. The mares belonged to 16 different breeds (Table 1), which were grouped into five different categories, according to the Breeding Association Regulations of the German Equestrian Federation from 2021.

**Table 1:** Categorisation and number of horse breeds, along with the corresponding number of parturitions

Category	Breeds	Number of parturitions	Total number
German riding horse	Warmblood	18	41
	Hanoverian horse	9	
	Thoroughbred	6	
	Oldenburger	3	
	Westphalian	2	
	Zweibrücker horse	1	
	Holsteiner	1	
	Trakehner horse	1	
Ponies and small horses	Pony	18	24
	Icelandic horse	4	
	Fjord horse	1	
	Haflinger	1	
Other breeds	Quarter Horse	3	7
	Friesian	2	
	Tinker Horse	1	
	Paint Horse	1	
Heavy Warmblood and Coldblood breeds		0	0
Trotting horse		0	0
Total	16	72	72

To diagnose dystocia, a standardised obstetric examination procedure was carried out, which was defined before the start of this study.

During the obstetric examination, the following vital parameters of the mares were first recorded by means of a general examination and were then recorded for a separate future study: State of consciousness, pulse and respiratory rates, rectal temperature, mucosal colour and capillary refill time.

The specialist obstetric examination began with an examination of the anogenital area. Attention was focused on oedema, injuries and discharge. During the subsequent manual vaginal and uterine examination, the condition of the soft birth canal was assessed. The following findings were recorded for the foetus:

- Presentation of the foetus: Anterior, posterior or transverse presentation.
- Position of the foetus: dorsal, lateral or ventral position.
- Posture of the foetus: extended posture or postural abnormalities of the head and/or limbs.
- Location of the foetus: describes the location of protruding, entering or exiting foetal parts in relation to the maternal pelvic cavity.
- Size of the foetus: normal size, absolute or relative foetopelvic disproportion.
- Whether the foetus was alive.

Only cases in which all the findings could be recorded were included in the analysis.

Treatment was initiated based on the findings. Manipulation and extraction (conservative obstetrics) as well as foetotomies were performed on a standing, sedated mare under epidural anaesthesia. Caesarean section was performed under general anaesthesia in dorsal recumbency in the *linea alba.*

Manipulation and extraction were selected when correction of presentation, position and posture was possible and the foetus was expected to pass through the birth canal. A caesarean section was selected for a living foetus, if it was not possible to correct presentation, position and posture or if the foetus could not pass the birth canal. Foetotomy was selected for a dead foetus, if it was not possible to correct presentation, position and posture or if the dead foetus could not pass the birth canal.

Information related to the duration of dystocia was not used for data collection because of insufficient data.

### Statistical analysis

The statistical software SAS 9.4 (SAS^®^ Institute Inc., 2013. Base SAS^®^ 9.4 Procedures Guide: Statistical Procedures, 2nd edition ed. Statistical Analysis System Institute Inc., Cary, NC, USA) was used for statistical analyses.

A binomial test for uniform distribution was performed to analyse whether there was a significant difference in the frequency of foetal and maternal causes of dystocia. Animals with maternal and foetal causes were excluded from the analysis. Therefore, three cases were excluded.

## Results

During the study period, 72 cases of dystocia were recorded in the clinic. Treatment was provided by foetotomy in 49 cases. There were 26 full foetotomies and 23 partial foetotomies. Extraction was performed ten times and caesarean section was performed in ten other cases. No obstetric procedures were performed in three cases. In these cases, the mares died before obstetric care was provided.

### Causes of dystocia

The 72 cases of dystocia were owing to 122 causes (Table 2). The total number of causes was larger than 72, as several pathological conditions sometimes occurred in an individual case. An isolated cause of dystocia was identified in 37 mares (51.4%) (Table 3), whereas 35 mares (48.6%) displayed a combination of two or more causes. Various manifestations of incorrect posture of the foetal forelimbs were most frequently involved in a case of dystocia (25.4%). The most common isolated cause of dystocia diagnosed was incorrect posture of the foetal forelimbs (18.9%). At 13.5% each, incorrect foetal presentation, absolute foetopelvic disproportion, incorrect foetal position and incorrect posture of the foetal hindlimbs were the second most common isolated causes. Incorrect posture of the foetal forelimbs and head was the most common combination of causes at 25.7%.

**Table 2:** Frequency of dystocia causes in the mare without subdivision into isolated and multiple

Cause of dystocia			Number of cases	%
Incorrect posture of the foetal forelimbs	Bilateral carpal flexion		12	
	Unilateral carpal flexion		6	
	Unilateral shoulder flexion		6	
	Unilateral elbow flexion		4	
	Bilateral shoulder flexion		3	
			31	25.4
Incorrect posture of the foetal head	Lateral deviation of the head		14	
	Ventral deviation of the head		8	
	Dorsal deviation of the head		1	
			23	18.9
Incorrect posture of the foetal hindlimbs	Bilateral hip flexion		9	
	Bilateral tarsal flexion		4	
	Unilateral hip flexion		2	
	Unilateral tarsal flexion		2	
			17	13.9
Incorrect foetal position	Lateral position		7	
	Ventral position		5	
			12	9.8
Incorrect foetal presentation	Ventrotransverse presentation		6	
	Ventro-Vertical presentation		3	
	Dorsotransverse presentation		3	
			12	9.8
Foetal malformations	Malformation of the head		5	
	Hydrocephalus		4	
	Arthrogryposis of the hindlimbs		1	
	Ankylosis of the cervical spine		1	
			11	9
Foetopelvic disproportion	Absolute foetopelvic disproportion		8	
	Relative foetopelvic disproportion		2	
			10	8.2
Uterine torsion intrapartum			3	2.5
Twin collision			2	1.6
Adhesions of uterus and peritoneum (on the abdominal suture of a previous operation)			1	0.8
Total			122	100

**Table 3:** Frequency of isolated causes of dystocia in the mare

Causes		Number of cases	%
Incorrect posture of the foetal forelimbs	Bilateral carpal flexion	5	
	Unilateral elbow flexion	1	
	Bilateral shoulder flexion	1	
		7	18.9
Incorrect posture of the foetal hindlimbs	Bilateral tarsal flexion	3	
	Bilateral hip flexion	2	
		5	13.5
Foetopelvic disproportion	Absolute foetopelvic disproportion	5	13.5
Incorrect foetal presentation	Ventrotransverse presentation	2	
	Dorsotransverse presentation	2	
	Ventro-Vertical presentation	1	
		5	13.5
Incorrect foetal position	Lateral position	3	
	Ventral position	2	
		5	13.5
Incorrect posture of the foetal head	Lateral deviation of the head	3	
	Dorsal deviation of the head	1	
		4	10.8
Uterine torsion intrapartum		3	8.1
Foetal malformations	Hydrocephalus	2	5.4
Twin collision		1	2.7
Total		37	100

The causes of dystocia were linked to the foetus in 95.1% (116/122) of the cases. Maternal causes of dystocia accounted for 4.9% (6/122) of the cases and were caused three times by a *uterine torsion intrapartum*, twice by relative foetopelvic disproportion and once by a postoperative adhesion of the uterus and peritoneum. Foetal causes were significantly more frequent origin of dystocia (95.7%) (95% confidence interval = [87.8%, 99.1%]) than maternal causes (4.4%) (95% confidence interval = [0.1%, 12.2%]) (*P* < 0.0001).

### Mare mortality

Nine mares were not discharged alive (Table 4). Three of these mares died before obstetric care was provided. Of these patients, two were euthanised owing to poor prognosis and one died during the examination. The fourth mare did not survive the caesarean section.

**Table 4:** Maternal mortality rate in relation to the obstetric procedures

Type of obstetric care		Died			Discharged alive
		Before obstetric care	During obstetric care	Before being discharged	
Partial foetotomy				3	20
Total foetotomy				1	25
Conservative obstetric care				1	9
Caesarean section			1		9
No obstetric care	Euthanasia	2			
	Died during initial examination	1			
Total		3	1	5	63
		9/72 12.5%			

The five remaining mares survived the obstetric procedures but died before they were discharged. Of these five animals, one mare was euthanised the day after conservative obstetrics. The other four mares were euthanised within the frame of foetotomy because of a poor prognosis. In three of these mares, uterine perforation was diagnosed after or during partial foetotomy. Mesenteric rupture was suspected after full foetotomy in the fourth mare. It was not possible to determine whether the injuries were owing to foetotomy or previous obstetric measures.

In relation to the obstetric procedures, the maternal mortality rate for a foetotomy was 8.2% (4/49), with 13% (3/23) as part of a partial foetotomy and 3.8% (1/26) after a full foetotomy. 10% (1/10) of the mares died after both conservative obstetric care and a caesarean section.

Six of the nine mares died in the presence of an isolated cause of dystocia. The remaining three mares had multiple causes of dystocia (Table 5).

**Table 5:** Mare mortality in relation to isolated and multiple causes of dystocia

Causes of dystocia		Died		
		Before obstetric care	During obstetric care	Before being discharged
Isolated causes	Bilateral tarsal flexion	1		1
	Lateral position			1
	Uterine torsion		1	
	Unilateral elbow flexion			1
	Ventrotransverse presentation	1		
Multiple causes	Bilateral hip flexion + unilateral tarsal flexion + lateral postion	1		
	Unilateral elbow flexion + absolute foetopelvic disproportion			1
	Ventrotransverse presentation + malformation of the head			1
Total		3	1	5
		9		

### Foal mortality

Owing to a twin pregnancy, 73 foetuses were delivered from 72 pregnancies. At presentation, the foetus was still alive in 18 cases and 55 foals were already dead before the start of veterinary treatment (Table 6). Seven foals were born alive. Two of these foals died within the first 24 h after birth. Three foals were euthanised before the mother was discharged, because of deformities. Two foals were discharged alive. In relation to the total number of births in which the foetus was alive at the start of obstetric care, the foetal mortality rate was 61.1% (11/18) and 88.9% (16/18) by the time the mother was discharged. Categorised according to the respective obstetric procedure performed, the foetal mortality rate for conservative obstetric care was 60% (6/10) and 80% (8/10) before the mare was discharged. Five of the eight foetuses were not delivered alive during the caesarean section. None of the eight foals that were alive at the start of the caesarean section could be discharged alive.

**Table 6:** Foetal mortality in relation to the obstetric procedures

Type of obstetric care	Died before obstetric care	Died		Discharged alive
		During obstetric care	Before being discharged	
Partial foetotomy	24			
Total foetotomy	26			
No obstetric care	3			
Conservative obstetric care		6	2	2
Caesarean section	2	5	3	
Total	55	11	5	2
		16/18 88.9%		

Twelve of the sixteen foals that died during obstetric care or before discharge had dystocia with isolated causes. Multiple causes were identified in the four remaining dystocias (Table 7).

**Table 7:** Foal mortality during obstetric care or before being discharged in relation to isolated and multiple causes of dystocia

Causes of dystocia		Died	
		During obstetric care	Before being discharged
Isolated causes	Uterine torsion	3	
	Bilateral hip flexion	2	
	Ventral position		2
	Ventrotransverse presentation	1	1
	Lateral position	1	
	Lateral deviation of the head		1
	Unilateral elbow flexion	1	
Multiple causes	Bilateral carpal flexion + ventral deviation of the head	1	
	Bilateral hip flexion + ventrotransverse presentation + chronic adhesive perimetritis	1	
	Bilateral shoulder flexion + ventro-vertical presentation		1
	Unilateral shoulder flexion + lateral deviation of the head	1	
Total		11	5
		16	

## Discussion

In the literature, there are a number of publications that investigate the causes of dystocia in horses, but they do not consider that different causes of dystocia can occur in combination, although this aspect is important to know in terms of treatment. In the present study, multiple disorders occurred almost as frequently as isolated causes and foetal causes of dystocia occurred more frequently than maternal causes. A number of renowned books describe the correction of isolated causes of dystocia [12–14]. However, no sequences are available for the treatment of multiple causes of dystocia. The aim of future studies will be to address this issue.

The most common cause of dystocia is incorrect posture of the foetus, particularly of the foal’s long extremities [15, 16]. In the present study, postural deviations of the forelimbs were the most common general (25.4%) and isolated (18,9%) cause of dystocia. This finding is not consistent with the findings of other studies [4, 9, 17], which reported an isolated incorrect foetal posture of the head and neck as the most common cause of dystocia in a specialist obstetric clinic. However, notably, in the present study foetal deviation of the head/neck posture was the second most common cause of dystocia. As an isolated cause of dystocia, it was determined to be the sixth most common cause after incorrect posture of the foetal hindlimbs, absolute foetopelvic disproportion, incorrect foetal presentation and incorrect foetal position.

Notably, the literature seldom discusses the combinations of causes for incorrect posture of the extremities. Two studies emphasized that incorrect posture of the head and forelimb also occurred in combination in their studies, but without stating the exact data [11, 18]. Only Frazer et al. [19] state in their survey of two obstetric clinics that the combination of causes of dystocia of incorrect posture of the foetal head and forelimbs was diagnosed in 15 out of 150 dystocia cases. In another study [1], a lower prevalence (4/106) was given for the occurrence of incorrect posture of the head and limbs as the only combination of causes. However, the aforementioned study was not conducted at an obstetric clinic with dystocia as the reason for admission, but in a facility for birth monitoring: therefore, it is only comparable to a limited extent. Vandeplassche [20] takes the view that dystocia cases in an obstetric clinic are mainly “complicated” cases and should be distinguished from dystocia in a veterinary practice. This fact has been reflected in our study, in that a large number of foetuses were already dead on presentation at the clinic and there was a high mortality rate even after a live foal was born.

The most common combination in the present study was an incorrect posture of the foetal head and forelimbs (25,7%). The second most combination was an incorrect posture of the foetal forelimbs with an absolute foetopelvic disproportion, occurred much less frequently, at 8.6%. In relation to all 72 cases of dystocia, incorrect posture of the head and forelimbs was diagnosed in 12.5% of cases. This confirms the results of the only comparable study with a slight deviation of 2.5% [19].

The large number of postural abnormalities demonstrates the importance of training veterinarians caring for broodmares to correct these abnormalities. In the opinion of the authors, the following procedure has proven successful regarding the combination of incorrect posture of the head and forelimb(s):

When a larger amount of space is required, the posture of the foetal head should be corrected first. Then, the forelimb(s) should be corrected while ensuring that the mucosa is protected.

Foetal causes of dystocia occurred more frequently than maternal causes in the present study. This confirms the prevalent findings of retrospective studies that equine dystocia is primarily of foetal origin [3, 8, 17]. A recent prospective study on dystocia in horses also found that the causes of dystocia were more often foetal than maternal [21].

The mares in the study population belonged to 16 different breeds. The number of parturitions assigned to each breed was considered too low to derive breed predispositions. However, 4 of 8 dystocia cases with absolute foetopelvic disproportion were ponies. Further studies with a larger number of cases are necessary to verify possible causality.

Nine mares were not discharged alive after dystocia. Differentiated according to the respective obstetric procedure, the mortality rate after a partial foetotomy was 13% and 3.8% after a total foetotomy. In contrast, a literature research showed a lower mortality rate in the period 1991–2021 for partial foetotomy (9%), compared with full foetotomy (27%) [6]. For this reason, the literature has so far attributed a higher risk of death for the mare after a full foetotomy [22]. However, in this context, some studies made no distinction between partial and full foetotomies and, imultaneously, showed similar average mortality rates as in the present study of 8.2% [20, 23]. The prevailing opinion, that full foetotomies inherently represent a greater risk of mortality in mares than partial foetotomies, should therefore be examined in future studies. A literature review of case studies for equine caesarean sections published between 1991 and 2021 showed an average maternal mortality of 14% [6], which is the same level as the data presented here. There is also only a slight deviation with regard to maternal mortality from one of the few publications in which conservative obstetric care was provided without general anaesthesia which mentions a mare mortality rate of 13.6% [4]. The present study has found a similar mare mortality rate of 10% (1/10).

Owing the small number of cases, no correlation between increased maternal or foetal mortality and the specific causes of dystocia could be inferred (Tables 5 and 7). Further studies are necessary to analyse this aspect.

With dystocia, the risk of a stillborn foal is ten times higher than with eutocia [24]. A comparable survey showed that following conservative or operative obstetric care, the foetal mortality rate was 95% up to discharge [4]. This figure is consistent with the foetal mortality rate in the present study, which at 88.9% (16/18) also points to a high foetal mortality rate before discharge. Significantly high foetal mortality rates have also been reported for caesarean sections. Based on 6 studies, an average foetal death rate of 75% (163/216) up until the time of discharge was determined for the period 1991–2021 [6]. The individual mortality rates vary from 69 to 96% and are therefore close to the foetal mortality rate of 100% in the present study. Comparable foetal mortality rates with respect to conservative obstetric care without general anaesthesia are not available. In the context of controlled vaginal delivery, in which the manipulation and extraction is performed on an anaesthetised mare, in the present study a mortality rate of 80% (8/10) was determined until the foals were discharged. Byron et al. [7] reported a similarly high mortality rate of 68% (120/177) at the time of discharge.

## Conclusions

Despite advances in veterinary medicine, foetal and maternal mortality in equine dystocia remains remarkably high compared with older studies in which data were collected in an obstetric clinic. As in other studies, fetal causes of dystocia occurred more frequently than maternal causes. In the present study, multiple disorders occurred almost as frequently as isolated causes. For the first time, the importance of at least two causes of dystocia was demonstrated simultaneously. Future studies should evaluate procedures for handling the multiple causes of equine dystocia.

## Data Availability

The data supporting this study’s findings can be made available from the corresponding author upon reasonable request.

## Declarations

### Prior publication

The data present herein have not been previously published.

## References

[1] McCue PM, Ferris RA. Parturition, dystocia and foal survival: a retrospective study of 1047 births. Equine Vet J 2012:44; Suppl 41:22–5. 10.1111/j.2042-3306.2011.00476.x 10.1111/j.2042-3306.2011.00476.x 22594021

[2] Lu KG, Barr BS, Embertson R, Dallap Schaer B. Dystocia—A true equine emergency. Clin Tech Equine Pract. 2006;5:145–53. 10.1053/j.ctep.2006.03.008.

[3] Ellerbrock M, Wehrend A. Definition, incidence and causes of dystocia in horses – a review of the literature. Tierarztl Prax Ausg G Grosstiere Nutztiere. 2023;51:22–34. 10.1055/a-2006-9248. 36913938 10.1055/a-2006-9248

[4] Freeman DE, Hungerford LL, Schaeffer D, Lock TF, Sertich PL, Baker GJ, et al. Caesarean section and other methods for assisted delivery: comparison of effects on mare mortality and complications. Equine Vet J. 1999;31:203–7. 10.1111/j.2042-3306.1999.tb03173.x. 10402132 10.1111/j.2042-3306.1999.tb03173.x

[5] Rioja E, Cernicchiaro N, Costa MC, Valverde A. Perioperative risk factors for mortality and length of hospitalization in mares with dystocia undergoing general anesthesia. Can Vet J. 2012;53:502–10. 23115362 PMC3327588

[6] Ellerbrock M, Wehrend A. Morbidity and mortality of mare and foal following dystocia – a literature review. Tierarztl Prax Ausg G Grosstiere Nutztiere. 2023;51:314–26. 10.1055/a-2180-2182. 37956674 10.1055/a-2180-2182

[7] Byron CR, Embertson RM, Bernard WV, Hance SR, Bramlage LR, Hopper SA. Dystocia in a referral hospital setting: approach and results: Approach and results. Equine Vet J. 2003;35:82–5. 10.2746/042516403775467405. 12553468 10.2746/042516403775467405

[8] Lanci A, Perina F, Donadoni A, Castagnetti C, Mariella J. Dystocia in the Standardbred Mare: A retrospective study from 2004 to 2020. Animals. 2022. 10.3390/ani12121486 10.3390/ani12121486 PMC9219446 35739823

[9] Carluccio A, Contri A, Tosi U, de Amicis I, de Fanti C. Survival rate and short-term fertility rate associated with the use of fetotomy for resolution of dystocia in mares: 72 cases (1991–2005). J Am Vet Med Assoc. 2007;230:1502–5. 10.2460/javma.230.10.1502. 17504042 10.2460/javma.230.10.1502

[10] Rosales C, Krekeler N, Tennent-Brown B, Stevenson MA, Hanlon D. Periparturient characteristics of mares and their foals on a New Zealand Thoroughbred stud farm. N Z Vet J. 2017;65:24–9. 10.1080/00480169.2016.1244021. 27705540 10.1080/00480169.2016.1244021

[11] Vandeplassche M. The pathogenesis of dystocia and fetal malformation in the horse. J Reprod Fertil Suppl. 1987;35:547–52. 3479608

[12] Parkinson TJ, Vermunt JJ, Noakes DE. Fetal Dystocia in Livestock: Delivery per vaginam (Chap. 14) In: Veterinary Reproduction and Obstetrics, 10th ed. Noakes DE, Parkinson TJ, England GCW, eds. Elsevier. 2018; 250–276.

[13] Vandeplassche M, Dystocia. Chap. 68. In: Mc Kinnon AO, Voss JL, editors. Equine Reproduction. Philadelphia: Lea and Febiger; 1993. pp. 578–87.

[14] Hospes R, Konservative Geburtshilfe. Geburtshilfe. Reproduktionsstörungen beim weiblichen Tier. Reproduktionsstörungen. In: Handbuch Pferdepraxis, 4. Aufl. Brehm W, Gehlen H, Ohnesorge B, Wehrend A, Hrsg. Stuttgart: Enke. 2017; 667–669; 2017.

[15] Blanchard TL, Morehead JP, Whitman JL, Peterson E. How to provide obstetrical intervention in equine ambulatory practice. Proc Amer Assoc Equine Pract. 2011;57:280–3.

[16] Mc Gladdery A. Dystocia and Postpratum complications in the mare. Pract. 2001;23:74–80. 10.1136/inpract.23.2.74.

[17] Karadjole T, Bačić G, Mačešić N, Karadjole M, Dobranić T, Makek Z, et al. Ursachen Von Dystokien Bei Stuten. Tierärztl Umsch. 2008;63:183–5.

[18] Leidl W, Stolla R, Schmid G. Zur Schwergeburt Bei Der Stute. I. Ursachen, konservative Geburtshilfe und Fetotomie. Tierärztl Umsch. 1993;48:408–12.

[19] Frazer GS, Perkins NR, Blanchard TL, Orsini J, Threlfall WR. Prevalence of fetal maldispositions in equine referral hospital dystocias. Equine Vet J. 1997;29:111–6. 10.1111/j.2042-3306.1997.tb01651.x. 9104559 10.1111/j.2042-3306.1997.tb01651.x

[20] Vandeplassche M. Selected topics in equine obstetrics. Proc Amer Assoc Equine Pract. 1992;38:623–8.

[21] Ellerbrock M, Krohn J, Büttner K, Wehrend A. Dystocia frequency and causes in horses with pregnancy disorders or a history of dystocia: a prospective study. Repro Domest Anim. 2024;59:e14541. 10.1111/rda.14541. 10.1111/rda.14541 38426354

[22] Stolla R, Leidl W, Schmid G. Zur Schwergeburt Bei Der Stute: II. Sectio Caesarea. Tierärztl Umsch. 1997;52:85–91.

[23] Wehrend A, Herfen K, Hospes R, Bostedt H. März. Die Fetotomie als geburtshilfliche Operation beim Pferd unter besonderer Berücksichtigung der puerperalen Behandlung - eine Follow-up Studie. DVG- Tagung der Fachgruppe Pferdekrankheiten, Wiesbaden, 16–17 2000; 278–281.

[24] Morley PS, Townsend HG. A survey of reproductive performance in Thoroughbred mares and morbidity, mortality and athletic potential of their foals. Equine Vet J. 1997;29:290–7. 15338910 10.1111/j.2042-3306.1997.tb03126.x

